# Precision Health–Enabled Machine Learning to Identify Need for Wraparound Social Services Using Patient- and Population-Level Data Sets: Algorithm Development and Validation

**DOI:** 10.2196/16129

**Published:** 2020-07-09

**Authors:** Suranga N Kasthurirathne, Shaun Grannis, Paul K Halverson, Justin Morea, Nir Menachemi, Joshua R Vest

**Affiliations:** 1 Center for Biomedical Informatics Regenstrief Institute Indianapolis, IN United States; 2 School of Medicine Indiana University Indianapolis, IN United States; 3 Richard M Fairbanks School of Public Health Indiana University Indianapolis, IN United States; 4 Eskenazi Health Indianapolis, IN United States

**Keywords:** social determinants of health, supervised machine learning, delivery of health care, integrated, wraparound social services

## Abstract

**Background:**

Emerging interest in precision health and the increasing availability of patient- and population-level data sets present considerable potential to enable analytical approaches to identify and mitigate the negative effects of social factors on health. These issues are not satisfactorily addressed in typical medical care encounters, and thus, opportunities to improve health outcomes, reduce costs, and improve coordination of care are not realized. Furthermore, methodological expertise on the use of varied patient- and population-level data sets and machine learning to predict need for supplemental services is limited.

**Objective:**

The objective of this study was to leverage a comprehensive range of clinical, behavioral, social risk, and social determinants of health factors in order to develop decision models capable of identifying patients in need of various wraparound social services.

**Methods:**

We used comprehensive patient- and population-level data sets to build decision models capable of predicting need for behavioral health, dietitian, social work, or other social service referrals within a safety-net health system using area under the receiver operating characteristic curve (AUROC), sensitivity, precision, F1 score, and specificity. We also evaluated the value of population-level social determinants of health data sets in improving machine learning performance of the models.

**Results:**

Decision models for each wraparound service demonstrated performance measures ranging between 59.2%% and 99.3%. These results were statistically superior to the performance measures demonstrated by our previous models which used a limited data set and whose performance measures ranged from 38.2% to 88.3% (behavioural health: F1 score *P*<.001, AUROC *P*=.01; social work: F1 score *P*<.001, AUROC *P*=.03; dietitian: F1 score *P*=.001, AUROC *P*=.001; other: F1 score *P*=.01, AUROC *P*=.02); however, inclusion of additional population-level social determinants of health did not contribute to any performance improvements (behavioural health: F1 score *P*=.08, AUROC *P*=.09; social work: F1 score *P*=.16, AUROC *P*=.09; dietitian: F1 score *P*=.08, AUROC *P*=.14; other: F1 score *P*=.33, AUROC *P*=.21) in predicting the need for referral in our population of vulnerable patients seeking care at a safety-net provider.

**Conclusions:**

Precision health–enabled decision models that leverage a wide range of patient- and population-level data sets and advanced machine learning methods are capable of predicting need for various wraparound social services with good performance.

## Introduction

### Background

The combination of precision health [1] and population health initiatives in the United States have raised awareness about how clinical, behavioral, social risk, and social determinants of health factors influence an individual’s use of medical services and their overall health and well-being [2]. Large-scale adoption of health information systems [3], increased use of interoperable health information exchange, and the availability of socioeconomic data sets have led to unprecedented and ever increasing accessibility to various patient- and population-level data sources. The availability of these data sets, together with a focus on mitigating patient social factors and uptake of machine learning solutions for health care present considerable potential for predictive modeling in support of risk prediction and intervention allocation [4,5]. This is particularly significant for wraparound services that can enhance primary care by utilizing providers who are trained in behavioral health, social work, nutritional counseling, patient navigation, health education, and medical legal partnerships in order to mitigate the effects of social risk and to address social needs [6].

Wraparound services focus on the socioeconomic, behavioral, and financial factors that typical medical care encounters cannot address satisfactorily [7,8], and when used, can result in improved health care outcomes, reduced costs [6,9], and better coordination of care. As such, these services are of significant importance to health care organizations that are incentivized by United States reimbursement policies to mitigate the effects of social issues that influence poor health outcomes and unnecessary utilization of costly services [10].

### Previous Work

In a previous study [11], we integrated patient-level clinical, demographic, and visit data with population-level social determinants of health measures to develop decision models that predicted patient need for behavioral health, dietitian, social work, or other wraparound service referrals. We also compared the performance of models built with and without population-level social determinants of health indicators. These models achieved reasonable performance with area under the receiver operating characteristic curve values between 70% and 78%, and sensitivity, specificity, and accuracy values ranging between 50% and 77%. We integrated these models into nine federally qualified health center sites operated by Eskenazi Health, a county-owned safety-net provider located in Indianapolis, Indiana. A subsequent trial identified increased rates of referral when predicted-need scores were shared with primary care end users [12]. Nevertheless, there were several limitations in our previous study such as limited patient-level measures, a level of aggregated data that was too coarse, poor optimization, lack of consideration of data temporality, and limited generalizability.

Our previous models included a wide range of patient-level clinical, behavioral, and encounter-based data elements as well as population-level social determinants of health measures; however, the models might have performed better with the inclusion of additional data elements such as medication data, insurance information, narcotics or substance abuse data, mental and behavioral disorders information inferred from diagnostic data, and patient-level social risk factors extracted from diagnostic data using ICD-10 classification codes [13].

Our previous use of population-level social determinants of health factors measured at the zip-code level did not contribute to any statistically significant performance improvements. A wider range of measures of social determinants of health captured at smaller geographic areas might have yielded more discriminative power and have led to significant performance improvements.

We used Youden J-index [14] which optimizes sensitivity and specificity to determine optimal cutoff thresholds; however, this resulted in poor precision (positive predictive values that ranged between 15% and 50%). Given the importance of optimizing precision, which represents a model’s ability to return only relevant instances, alternate optimization techniques should be used.

Our previous models included all data captured during the period under study, and not exclusively data elements that occurred prior to the outcome of interest. Failing to omit data elements that occurred after the outcomes of interest may have influenced the performance of these decision models [15].

We developed our previous approach using data that was extracted from a homegrown electronic health record system [16]. This limited its ability to be replicated across other settings that could support other widely used commercial electronic health record systems. Since our previous study, Eskenazi Health has transitioned to a commercial electronic health record system enabling us to adapt our solution to be vendor neutral and applicable to any electronic health record system.

### Objective

This study addressed the aforementioned limitations by using additional patient- and population-level data elements as well as more advanced analytical methods to develop decision models to identify patients in need of referral to providers that can address social factors. We evaluated the contribution of these enhancements by recreating the original models that had been developed during the previous study (phase 1) and comparing their performance to that of new models developed during this study (phase 2). Furthermore, during each phase, we evaluated the contribution of small-area population-level social determinants of health measures to improving model performance.

## Methods

### Patient Sample

We included adults (18 years of age or older) with at least one outpatient visit at Eskenazi Health between October 1, 2016 and May 1, 2018.

### Data Extraction

Primary data sources for the patient cohort were Eskenazi Health’s Epic electronic health record system and the statewide health information exchange data repository known as the Indiana Network for Patient Care [17], which provided out-of-network encounter data from hospitals, laboratory systems, long-term care facilities, and federally qualified health centers across the state. These data were supplemented with population-level social determinants of health measures derived from the US Census Bureau, the Marion County Public Health Department vital statistics system, and various community health surveys.

### Feature Extraction

To recreate the models developed during phase 1, we extracted a subset of features that had been used to train the original models [11]. We also extracted additional features for phase 2 enhancements. Table 1 presents an outline of the feature sets for each phase of model development.

**Table 1:** Comparison of the patient- and population-level data sets that were used for each phase.

Feature type	Phase 1	Added in phase 2
Demographics	Age, ethnicity, and gender	Insurance (Medicare, Medicaid, self-pay)
Weight and nutrition	None	BMI, hemoglobin A_1c_
Encounter frequency	Outpatient visits, emergency department encounters, and inpatient admissions	None
Chronic conditions	20 most common chronic conditions [18]	None
Addictions and narcotics use	Tobacco and opioid use	Alcohol abuse, opioid overdose, use disorders
Medications	None	145 categories of medication (categorized by therapeutic and pharmaceutical codes) [19]
Patient-level social risk	None	12 patient-level measures [20]
Population-level social determinants of health	48 social determinants of health measures [11]	60 social determinants of health measures [20]

### Preparation of the Gold Standard

We sought to predict the need for referrals to behavioral health services, dietitian counseling, social work services, and all other wraparound services, which included respiratory therapy, financial planning, medical-legal partnership assistance, patient navigation, and pharmacist consultations. We used billing, encounter, and scheduling data extracted from the Indiana Network for Patient Care and Eskenazi Health to identify patients who had been referred to supplementary services between October 1, 2016 and May 1, 2018. We assumed that a patient with a referral had been in need of that referral even if the patient subsequently canceled or failed to keep the appointment.

### Data Vector Preparation

We prepared two data vectors for each wraparound service for phase 1 modeling—a clinical data vector consisting of only patient-level data elements and a master data vector consisting of both patient- and population-level elements. Next, we created two more data vectors for each wraparound service for phase 2 data—a clinical data vector consisting of only patient-level data elements and a master data vector consisting of both patient- and population-level elements. For each patient, we included only data for events that had occurred at least 24 hours prior to the final outcome of interest. Features such as age (discrete by whole years); weight- or nutrition-based (categorical); gender (categorical); ethnicity (categorical); encounter frequency (number of each type per patient); and addictions or use of narcotics, chronic conditions, medications, and patient-level social risk (binary indicating presence or absence).

Population-level social determinants of health measures were categorized into three groups—socioeconomic status, disease prevalence, and other miscellaneous factors (such as data on calls made by those who were seeking public assistance). Measures that were reported from across 1150 census tracts were used to calculate *z* scores (a numerical measurement relating a given value to the mean in a group of values) for each of the three categories. The *z* scores were grouped into clusters using the *k*-means algorithm [21] and the elbow method [22].

As requested by dietitians who consulted on our efforts, for dietitian referrals, prediction of need was restricted to a subset of patients with specific risk conditions (Multimedia Appendix 1). Thus, data vectors for dietitian referrals included only patients with one or more of these conditions, which were identified by ICD-10 classification codes.

### Machine Learning Process for Phase 1 Models

We randomly split each data vector into groups of 80% (training and validation data set) and 20% (test set). We replicated the same processes that were used during phase 1 [11] to recreate a new set of models to be used for comparison.

### Machine Learning Process for Phase 2 Models

We split each data vector into random groups of 80% (training and validation data set) and 20% (test set). We applied randomized lasso-based [23] feature selection to the 80% training and validation data set to identify the most relevant features for each outcome of interest. We used machine learning in Python (version 3.6.1; scikit-learn library, version 0.21.0) [24] to build extreme gradient boosting [25] classification models to predict the need for referrals. The extreme gradient boosting algorithm is an implementation of gradient boosted decision trees [26] designed for speed and performance. It has demonstrated a strong track record of outperforming other decision trees and other classification algorithms in machine learning competitions [27]. The extreme gradient boosting algorithm consisted of multiple parameters, each of which could affect model performance. Thus, we decided to perform hyperparameter tuning on the training and validation data set using randomized search and 10-fold cross-validation. Decision model parameters that were modified as part of the hyperparameter tuning process are listed in Multimedia Appendix 2. The best performing models, parameterized using hyperparameter tuning, were applied to the test data sets.

### Analysis

**Figure 1:**
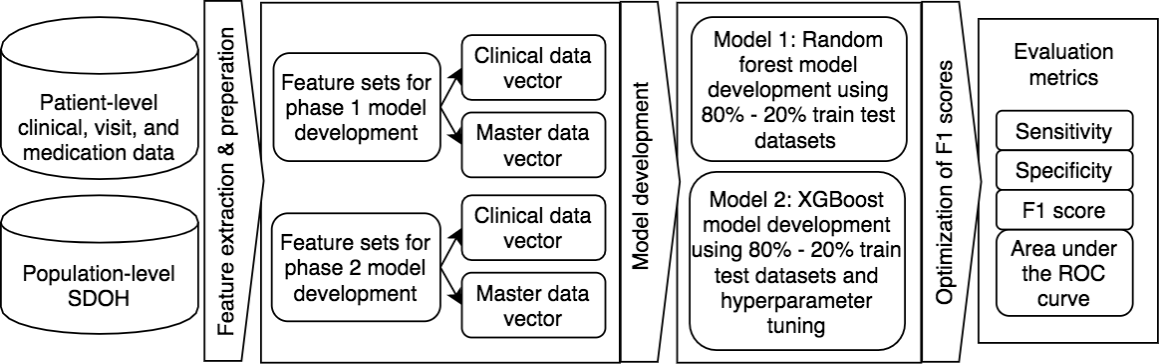
The complete study approach from data collection and decision-model building to evaluation of results. ROC: receiver operating characteristic; SDOH: social determinants of health; XGBoost: extreme gradient boosting.

We assessed the performance of each decision model using the test set. For each record in the test set, each decision model produced a binary outcome (referral needed or referral not needed) and a probability score. We used these scores to calculate area under the receiver operating characteristic curve (AUROC), sensitivity, precision, F1 score, and specificity for each model. These measures were calculated using thresholds that optimized sensitivity and precision scores. We also calculated 95% confidence intervals for each measure using bootstrap methods [28]. *P* values were calculated using guidelines presented by Altman and Bland [29]. *P* values<.05 were deemed statistically significant. For the models trained during each phase, we evaluated the contribution of population-level measures by comparing the performance of models trained using master (with population-level measures) vector models to the performance of clinical (without population-level measures) vector models. Next, we evaluated the value of the additional data sets and analytical methods that were used to train phase 2 models by comparing their performance to that of models trained in phase 1. Figure 1 presents a flowchart that describes the approach.

## Results

Our patient sample consisted of 72,484 adult patients (Table 2). Of these patients, 15,867 (21.9%) met the dietitian referral criteria. Similar to that of phase 1, our patient population reflected an adult, urban, low-income primary care safety-net population; patients ranged in age from 18 to 107 years and were predominantly female (47,187/72,484, 65.1%). Referral types, which constituted our gold standard reference, were behavioral health (12,162/72,484, 16.8%), social work (4104/72,484, 5.7%), dietitian counseling (4330/15,867, 27.3%), and other services (17,877/72,484, 24.7%).

As with our previous effort, use of population-level social determinants of health measures led to only minimal changes in each performance metric across models trained under phases 1 and 2, and were not statistically significant (behavioural health: F1 score *P*=.08, AUROC *P*=.09; social work: F1 score *P*=.16, AUROC *P*=.09; dietitian: F1 score *P*=.08, AUROC *P*=.14; other: F1 score *P*=.33, AUROC *P*=.21). Thus, we evaluated the contribution of the additional data sets, classification algorithms, and analytical approaches leveraged in phase 2 by comparing clinical vector models developed during phase 1 to those developed during phase 2.

Table 3 presents a comparison of clinical vector model performance for phase 1 and phase 2. Phase 2 models yielded significantly better results than those of phase 1 models across all performance metrics except sensitivity for social work services (phase 1: 67.0%, 95% CI 63.4%-72.2%; phase 2: 72.4%, 95% CI 69.1%-75.6%; *P*=.07). Phase 2 decision models reported performance measures ranging from 59.2% to 99.3% which were statistically superior to performance measures reported by phase 1 models which ranged from 38.2% to 88.3%. For every clinical vector, phase 2 models reported significantly better area under the receiver operating characteristic curve values than those reported for phase 1 models (behavioral health: *P*=.01; social work: *P*=.03; dietitian: *P*=.001; other: *P*=.02). Furthermore, phase 2 precision scores were significantly greater than those reported in phase 1 (behavioral health: *P*<.001; social work: *P*<.001; dietitian: *P*=.02; other: *P*<.001). We also evaluated model fit using logarithmic loss (log loss), which measures the performance of a classification model where prediction input is a probability between 0 and 1, and using lift curves [30], which compares a decision model to a random model for the given percentile of top scored predictions. Log loss values were 0.09 (behavioral health), 0.07 (social work), 0.32 (dietitian), and 0.34 (other). Lift scores for each decision model are shown in a figure in Multimedia Appendix 3.

**Table 2:** Characteristics of the adult, primary care patient sample whose data were used in phase 2 risk predictive modeling.

Demographic characteristics		Values
Age (years), mean (SD)		44.1 (16.6)
**Gender (N=72,484), n (%)**		
	Male	25,297 (34.9)
	Female	47,187 (65.1)
**Insurance provider (N=72,484), n (%)**		
	Medicaid or public insurance	41,316 (57.0)
	Private	31,168 (43.0)
**BMI category (N=72,484), n (%)**		
	BMI<18.5	6379 (8.8)
	18.5≤BMI<25	8698 (12.0)
	25≤BMI<30	10,148 (14.0)
	BMI≥30	20,875 (28.8)
	Missing	26,384 (36.4)
**Ethnicity (N=72,484), n (%)**		
	White, non-Hispanic	18,266 (25.2)
	African American, non-Hispanic	34,575 (47.7)
	Hispanic	15,149 (20.9)
	Other	4494 (6.2)

**Table 3:** Comparison of clinical vector model performance for phase 1 and phase 2.

Clinical vector performance measures		Model performance, % (95% CI)					
			Phase 1		Phase 2		*P* value^a^
**Behavioral health services**							
	Sensitivity		70.2 (68.0, 72.5)		86.3 (83.1, 88.9)		<.001
	Specificity		78.5 (78.0, 78.9)		99.1 (98.5, 99.7)		<.001
	F1 score		56.6 (53.6, 58.9)		90.4 (87.4, 93.4)		<.001
	Precision (positive predictive value)		47.4 (44.2, 49.6)		95.0 (92.0, 98.3)		<.001
	AUROC^b^		88.3 (87.4, 89.2)		98.0 (97.6, 98.5)		.01
**Social work services**							
	Sensitivity		67.0 (63.4, 72.2)		72.4 (69.1, 75.6)		.07
	Specificity		79.6 (79.1, 79.8)		99.3 (99.2, 99.6)		<.001
	F1 score		48.6 (45.0, 52.5)		82.5 (79.7, 85.3)		<.001
	Precision (positive predictive value)		38.2 (34.8, 41.2)		95.8 (93.8, 97.8)		<.001
	AUROC		87.6 (86.1, 89.2)		93.7 (92.5, 95.0)		.03
**Dietitian counseling services**							
	Sensitivity		60.7 (56.5, 64.7)		73.6 (70.5, 77.0)		.02
	Specificity		73.2 (71.9, 74.9)		93.3 (90.8, 94.6)		<.001
	F1 score		61.5 (57.3, 66.0)		76.4 (73.3, 80.4)		.001
	Precision (positive predictive value)		62.2 (58.1, 67.4)		79.4 (76.4, 84.2)		.02
	AUROC		82.5 (81.5, 83.6)		91.5 (90.3, 92.6)		.001
**Other wraparound services**							
	Sensitivity		44.5 (42.7, 46.1)		59.2 (56.5, 63.8)		.002
	Specificity		78.5 (77.5, 79.3)		92.9 (89.7, 96.1)		<.001
	F1 score		43.2 (40.0, 45.7)		65.5 (62.9, 67.6)		.01
	Precision (positive predictive value)		41.9 (37.7, 45.2)		73.4 (70.5, 77.7)		<.001
	AUROC		77.2 (76.2, 78.1)		85.3 (84.4, 86.0)		.02

^a^*P* values were calculated using confidence intervals [29].

^b^AUROC: area under the receiver operating characteristic curve.

## Discussion

### Principal Findings

Our study expanded upon our previous efforts to demonstrate the feasibility of predicting the need for wraparound services such as behavioral health, dietitian, social work and other services using a range of readily available patient- and population-level data sets that represent an individual’s well-being as well as their socioeconomic environment. Specifically, we demonstrated that inclusion of additional patient-level data sets that represented medication history, addiction and mental disorders, and patient-level social risk factors, as well as use of the extreme gradient boosting classification algorithm and advanced analytical methods for model development led to statistically superior performance measures. Furthermore, improved precision scores were made possible by additional data elements and alternate optimization techniques that maximized precision and recall scores and which greatly improved the practical application of our solution. Each decision model reported area under the receiver operating characteristic curve scores from 85% to 98%, which are superior to the global performance of prediction models on mortality [31], hospital readmissions [4], and disease development [32]; however, inclusion of additional population-level aggregate social determinants of health measures in our low-income population did not contribute significantly toward performance improvements despite the introduction of additional indicators, more granular geographic measurement units (by switching from zip code to census tract level), and vectorization methods that converted these to standardized scores to emphasize variance and create indices.

The inability of population-level social determinants of health measures to improve model performance may be because our patient population was comprised of an urban safety-net group with relatively little variability in socioeconomic, policy, and environmental conditions. Thus, it is possible that machine learning studies using larger, more diverse populations may benefit from the use of population-level data [33]. Moreover, the lack of improvement may be related to our choice of prediction outcome. Wraparound service providers work to address the social needs and risk factors of individual patients and not population-level social determinants. Likewise, social determinants of health factors influence social risk [34], but these population conditions are not the reason for referral to a wraparound service provider. It is likely that social factors are more relevant to, and observed by, the referring provider. Nevertheless, the continued lack of meaningful contribution to our models prompts questions regarding how to best leverage aggregate social determinants of health measures for decision making. This is an important and unanswered question, as census-based aggregate measures are the most widely available and easily accessible indicators of social determinants of health available to researchers and health organizations [35]. In contrast, several patient-level social and behavioral factors measures were influential in the models. This indicates the need for more widespread use and collection of social factors in clinical settings [36]. Electronic health record organizations seeking to identify patients with social risk factors and in need of social services must integrate the collection of social risk data into their workflow [37].

### Limitations

This work has limitations. Notably, the phase 2 model development approach leveraged the same urban safety-net population that was used to develop phase 1 models. Thus, though the phase 2 demonstrate superior performance, the results may not be generalizable to other commercially insured or broader populations. In addition, we only leveraged structured data that had been extracted from the Indiana Network for Patient Care or from Eskenazi Health for the machine learning process. These methods may not be utilized at other health care settings that are not part of a large, robust health information exchange. Expanding our approaches to different geographic regions would require standardization of population-level sources as well as infrastructure and interoperability measures to effectively store and exchange such data sets [38]. Also, we did not utilize any unstructured data sets for machine learning. This is a significant issue as up to 80% of health data may be collected in an unstructured format [39,40]. Despite these limitations, the considerable performance enhancements demonstrated by these models suggest significant potential to enable access to various social services; however, it must be noted that social determinants of health risk factors are often confounded with one another. Thus, mitigating a social need that arises from several social determinants of health risk factors may not result in any positive improvements to a patient [41].

### Future Work

Our next steps include expanding our models to predict additional wraparound services of interest. Furthermore, we believe that there is an acute need to improve the explainability and actionability of machine learning predictions using novel methods such as counterfactual reasoning [42]. We perceive that similar predictive models for minors and the services available to these patients would be of significant value for health care decision making. Our inability to utilize unstructured data sets for machine learning is a significant concern. Various natural language processing toolkits can leverage unstructured data sets for machine learning; however, integrating these toolkits into inproduction systems is challenging due to infrastructure and maintenance costs. Moreover, searching and indexing the massive quantities of free-text reports that are collected statewide would require additional computational effort, and may significantly increase computation time. We are currently engaged in efforts to utilize the Regenstrief Institute’s nDepth tool [43] to evaluate the ability to extract actionable elements at a production setting.

### Integration Into Electronic Health Record Systems

**Figure 2:**
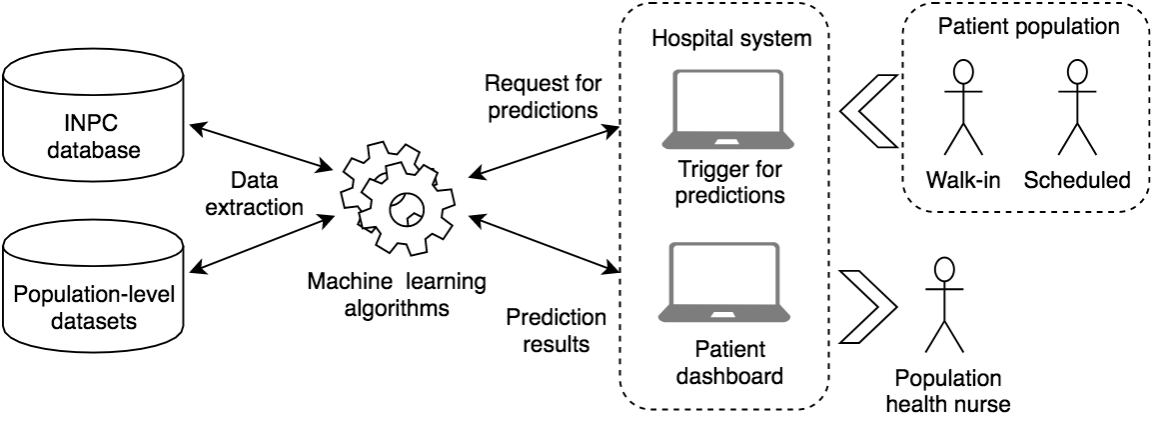
Integration of decision models into hospital workflow. INPC: Indiana Network for Patient Care.

As noted, this work built upon existing risk prediction efforts. We have integrated the updated decision models into the existing platform for all scheduled and walk-in appointments. Model results are presented to end users using a customized interface within the electronic health record with metadata on which features drove the extreme gradient boosting decision-making process, and with predicted probabilities categorized as low, rising, or high risk [12] (Figure 2). This study’s methodological work sets the foundation for our future evaluations of our intervention’s impact on patient outcomes.

### Conclusions

This study developed decision models that integrate a wide range of individual and population data elements and advanced machine learning methods that are capable of predicting need for various wraparound social services; however, population-level data may not contribute to improvements in predictive performance unless they represent larger, diverse populations.

## Appendix

Multimedia Appendix 1 List of risk conditions used to identify a subpopulation for predicting dietitian referrals.

Multimedia Appendix 2 Parameters that were modified as part of the hyperparameter tuning process for phase 2.

Multimedia Appendix 3 Lift scores reported by each decision model.

## Abbreviations

AUROC: area under the receiver operating characteristic curve
ICD-10: International Statistical Classification of Diseases, 10th Revision
