# Supplementary material for: Precision Health–Enabled Machine Learning to Identify Need for Wraparound Social Services Using Patient- and Population-Level Data Sets: Algorithm Development and Validation
Source: JMIR Med Inform. 2020 Jul 9;8(7):e16129. doi: 10.2196/16129 (PMC7380999; doi:10.2196/16129)
Supplement: Multimedia Appendix 1 [file medinform_v8i7e16129_app1.docx]

- Diabetes
- Eating Disorders (Anorexia, Bulimia)
- Elimination diets
- Food allergies
- GI diagnosis (Celiac, IBS, IBD, cirrhosis, etc.)
- Hypoglycemia
- Oncology
- Pregnancy
- Unintentional weight loss/ inadequate intake
- Vitamin or mineral deficiencies
