# Supplementary material for: Precision Health–Enabled Machine Learning to Identify Need for Wraparound Social Services Using Patient- and Population-Level Data Sets: Algorithm Development and Validation
Source: JMIR Med Inform. 2020 Jul 9;8(7):e16129. doi: 10.2196/16129 (PMC7380999; doi:10.2196/16129)
Supplement: Multimedia Appendix 2 [file medinform_v8i7e16129_app2.docx]

| Hyperparameter | Description | Range of parameters tested |
| --- | --- | --- |
| Number of estimators | Number of trees | Increments of 50 between 50 and 1000 |
| Minimum child weight | Minimum sum of weights of all observations required in a child | 1, 3, 5, 7, 9 |
| Gamma value | the minimum loss reduction required to split a node | 0.5, 1, 1.5, 2, 5 |
| Subsample | Fraction of observations to be randomly samples for each tree | 0.6, 0.8, 1.0 |
| Col sample by tree | Fraction of columns to be randomly samples for each tree | 0.6, 0.8, 1.0 |
| Max depth | Maximum depth of each tree | 3, 4, 5, 7 |
