# Supplementary figures and images for: Precision Health–Enabled Machine Learning to Identify Need for Wraparound Social Services Using Patient- and Population-Level Data Sets: Algorithm Development and Validation
Source: JMIR Med Inform. 2020 Jul 9;8(7):e16129. doi: 10.2196/16129 (PMC7380999; doi:10.2196/16129)

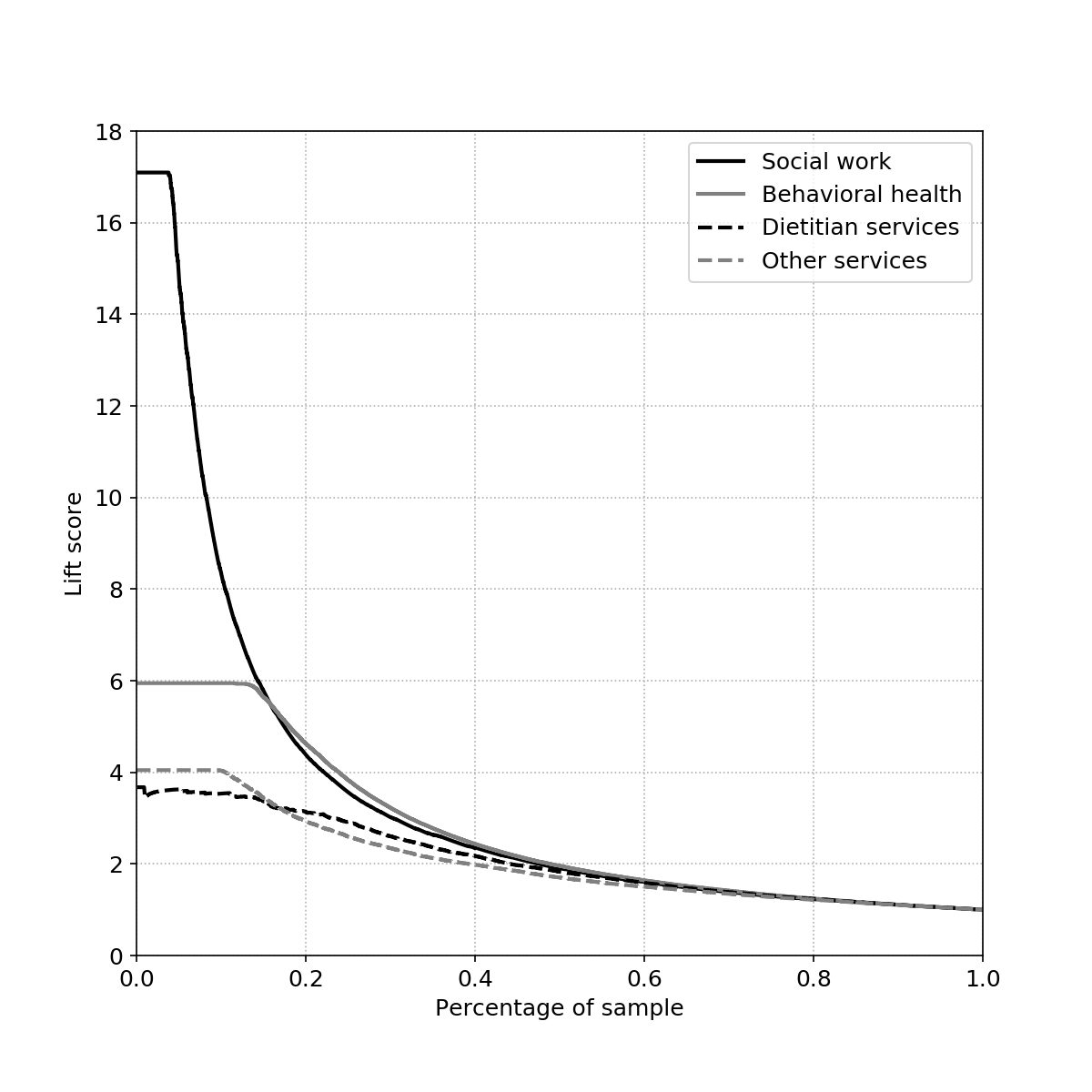

Supplement: Multimedia Appendix 3 [file medinform_v8i7e16129_app3.png]
